# Oral, not gut microbiota diversity, reflects the inflammation and neoplasia in patients with uveitis and vitreoretinal lymphoma

**DOI:** 10.1186/s12348-025-00517-2

**Published:** 2025-08-21

**Authors:** Michaela Brichova, Lucie Dlouha, Marketa Tenglerova, Johana Rehakova, Martin Kostovcik, Katerina Benesova, Stepan Coufal, Eliska Pivrncova, Zuzana Jiraskova Zakostelska, Miloslav Kverka, Eva Skrlova, Petra Svozilkova, Aneta Klimova, Klara Kostovcikova, Marek Trneny, Jarmila Heissigerova

**Affiliations:** 1 Department of Ophthalmology, 1 st Faculty of Medicine, Charles University, General University Hospital, Prague, Czech Republic; 2 1 st Department of Medicine, 1 st Faculty of Medicine, Charles University, General University Hospital, Prague, Czech Republic; 3 Laboratory of Cellular and Molecular Immunology, Institute of Microbiology, Czech Academy of Sciences, Prague, Czech Republic; 4 Laboratory of Fungal Metabolism and Genetics, Institute of Microbiology, Czech Academy of Sciences, Prague, Czech Republic

**Keywords:** Microbiome, Microbiota, Uveitis, Vitreoretinal lymphoma, Sequencing

## Abstract

**Purpose:**

Dysregulation of the microbiota on different mucosal surfaces is associated with both immune-mediated and malignant diseases. Nevertheless, the involvement of different microbial communities is still poorly characterized. The aim of our study was to compare oral and gut microbiota composition between patients with uveitis, vitreoretinal lymphoma (VRL), and controls.

**Methods:**

This study was designed as a prospective observational study. The inclusion criteria were treatment-naïve patients with immune-mediated uveitis or newly diagnosed VRL. The buccal swab and faecal samples were collected and bacterial 16S ribosomal RNA gene sequencing was used to identify the oral and gut microbiota.

**Results:**

We enrolled 18 patients with uveitis, median age 39 years, 16 patients with VRL, median age 67.5 years, and 16 controls, median age 63 years. In the oral microbiota, the patients suffering from uveitis showed significant enrichment of genera *Pseudomonas* (*p* < 0.0001 and *p* < 0.0001), and *Diaphorobacter* (*p* = 0.007 and 0.013) and reduction of *Streptococcus* (*p* < 0.0001 and *p* < 0.0001) when compared to patients with VRL and control subjects, respectively. In addition, these patients had also significantly higher relative abundance of the genus *Enhydrobacter* (*p* = 0.029) and lower abundance of the genera *Gemella* (*p* = 0.002), *Neisseria* (*p* = 0.008), and *Prevotella* (*p* = 0.011) when compared to patients with VRL. We found only minor changes in the gut microbiota.

**Conclusion:**

Our study, as the first one, highlighted significant differences in the composition of oral microbiota among patients with uveitis, VRL, and control subjects.

## Introduction

The human microbiota has attracted the attention of many medical specialities, including ophthalmology. Aberrant microbial composition or function, known as dysbiosis, has been linked to various autoimmune and autoinflammatory diseases, including inflammatory bowel disease [1–3], allergies [4], uveitis [5], and cancer [6]. However, the role of site-specific and distant microbiome in the disease pathogenesis has not yet been satisfactorily clarified.

Only a small fraction, specifically about 30%, of uveitides can be attributed to identifiable infectious agents [7]. In such cases, targeted therapy is indicated by pathogen detection (PCR, serology) or by a characteristic clinical picture. Most uveitides are non-infectious and belong to the immune-mediated inflammatory disorders (IMID). Approximately 25–30% of non-infectious intraocular inflammations are part of some systemic disease (e.g. ankylosing spondylarthritis, juvenile idiopathic arthritis, sarcoidosis, multiple sclerosis, Behcet’s disease) [8, 9]. Vitreoretinal lymphomas (VRL) are disorders that can mimic uveitis with their clinical picture and are the most common malignant uveitic masquerade syndromes [10]. Vitreoretinal lymphomas are formed by Large B-Cell Lymphomas (LBCL) and together with primary central nervous system (CNS) lymphoma (PCNSL) belong to a group of immune privilege sites lymphomas (IP-LBCL) [11, 12].

The experimental animal studies showed that severity of ocular inflammation can be modulated by microbiota depletion, i.e. germ-free or antibiotic-treated models [13–16], or by enriching the gut microbial community with different probiotics [17, 18]. To date, only a few observational studies comparing the gut microbiota in patients with uveitis and healthy controls have been published. One direction of the research deals with the role of gut microbiota in Human leukocyte antigen B27 (HLA-B27) associated diseases, including acute anterior uveitis [19–21]. The other one focuses on the gut microbiota in patients with Behcet’s disease (BD), birdshot chorioretinopathy, Vogt-Koyanagi-Harada syndrome (VKH), patients with anterior uveitis, or other types of uveitis [22–28].

Compared to uveitis research, the number of published studies investigating the gut microbiota in experimental animal models of lymphomas is substantially lower [29, 30]. Several studies on patients with systemic lymphoma have suggested that gut microbiota influences the occurrence and severity of the disease and affects the success of the therapy [31–36]. Nevertheless, the data on the microbiota of patients with systemic lymphoma are limited and there is no study on microbiota composition and function in patients with IP-LBCL and VRL specifically.

Microbial traits resulting in uveitis or VRL include dysbiosis, especially reduction of short-chain fatty acids (SCFA) producing strains, increasing the permeability of barriers. Translocation of whole microbes or their components/products promotes production of pro-inflammatory mediators and can serve as an antigen or self-antigen leading to chronic inflammation in specific sites [37]. The close proximity of the oral microbiota may influence the uveitis development as has been shown in few evidence-based studies [38–42]. Moreover, the role of oral microbiota is still insufficiently studied in uveitis as well as in different types of lymphomas. Therefore, our study is focused on examining the differences in the oral and gut microbiota composition among patients with uveitis, VRL, and control subjects.

## Methods

This study was designed as a prospective observational study. The inclusion criteria were treatment-naïve patients with immune-mediated uveitis or newly diagnosed VRL, who were enrolled during regular appointments at the Department of Ophthalmology at the General University Hospital in Prague. The diagnosis of VRL is based on cytology and flow cytometry or by haemato-oncological examination as published earlier [43]. The control subjects were recruited from healthy age-matched individuals. The exclusion criteria for enrolment comprised of infectious uveitis, HLA-B27 positivity, BD, recent antibiotic treatment (two months or less before sampling), and a history of intestinal disease or major intestinal resection. Samples for microbiota analysis were collected before any systemic treatment for uveitis or lymphoma. In addition, buccal swab was performed in the morning before brushing teeth, and before meals, except for water. Faecal samples were frozen within 5 h after collection and buccal swabs were collected using a sterile flocked swab (Dispolab, Czech Republic; Cat# 1646) and immediately immersed into a tube with sterile SCF-1 buffer (50 mM Tris–HCl, pH 7.5; 1 mM EDTA, pH 8.0; 0.5% Tween-20). All samples were stored at −80 °C until the DNA extraction.

Total DNA from faecal samples was extracted using ZymoBIOMICS DNA Miniprep Kit (ZYMO Research, USA; Cat# D4300) with repeated bead-beating using FastPrep homogenizer (MP Biomedicals). The extraction of DNA from buccal swabs was performed by the DNeasy PowerBiofilm kit (Qiagen, Germany; Cat# 24000-50) with minor modifications to the protocol as previously described [44]. The DNA was then quantified using Qubit dsDNA High Sensitivity kit (Thermo Fisher Scientific, USA; Cat# Q32853). PCR targeting V3 and V4 regions of bacterial 16S was conducted using Kapa HiFi DNA polymerase (Kapa Biosystems, USA; Cat# KK2602) and primers 341 F 5′-CCTACGGGNGGCWGCAG-3′ and 806R 5′-GGACTACHVGGGTWTCTAAT-3′ (Generi Biotech, Czech Republic) [45]. Cycling conditions consisted of initial denaturation (94 °C, 3 min), followed by 30 cycles of denaturation (94 °C, 30 s), annealing (54.2 °C, 45 s) and extension (72 °C, 75 s), and final extension (72 °C, 10 min). PCR triplicates were pooled and purified by SequalPrep Normalization Plate Kit (Thermo Fisher Scientific, USA; Cat# A1051001). Samples within library were pooled and sequencing adaptors were ligated using TruSeq DNA PCR-free LT Sample Preparation Kit (Illumina, USA; Cat# FC-121-3001). Ligated libraries were quantified with KAPA Library Quantification Kit (Kapa Biosystems, USA; Cat# KK4824) and sequenced on MiSeq Illumina Platform using Miseq Reagent Kit v3 (Illumina, USA; Cat# MS-102-3003) at The Genomics Core Facility, CEITEC (Brno, Czech Republic).

Sequencing data were processed using QIIME version 1.9.1 [46]. Quality filtering, chimera detection and read demultiplexing and clustering were done as described previously [3]. Identification of representative sequences was done using RPD classifier [47] against bacterial GREENGENES database 13.8 [48]. Finally, operational taxonomic units (OTUs) table was produced.

For microbiome analysis, the Shannon, Chao1 and Faith Phylogenetic Diversity (PD) indices were used to describe the alpha diversity. The relative abundance of microbes was used for beta diversity description. We explored variation in microbial abundances using principal component analysis (PCA) using packages FactoMineR (ver. 2.9) [49], factoextra (ver. 1.0.7) [50], and visualized by ggplot2 package (ver. 3.4.4) [51] in R (ver. 4.3.0). Systematic differences in microbial profiles among patient groups were tested by redundancy analysis (RDA) using package vegan (ver. 2.6-4) [52]. To eliminate the effect of extreme values, abundances were square root transformed and scaled prior to PCA and RDA analyses.

Data were analysed using GraphPad Prism version 8.4.3 for Windows (GraphPad Software, San Diego, CA, USA; www.graphpad.com). Statistical differences were calculated by nonparametric Kruskal-Wallis test with Dunn´s post-hoc testing or two-way ANOVA with Tukey´s multiple comparisons test. Data were expressed as medians. Differences were considered statistically significant at *p* ≤ 0.05.

## Results

### Cohorts

In the period 2019–2022, we have enrolled 18 patients with uveitis (Table 1): 4 men, 14 women, median age 39 years (26–68 years); 16 patients with VRL (Table 2): 11 men, 5 women, median age 67.5 years (42–83 years); and 16 controls (4 men, 12 women), median age 63 years, (29–71 years).

**Table 1:** Cohort description – patients with uveitis

Patient	F/M	Age at microbiome sample collection (years)	Type of uveitis	Laterality
1	F	39	anterior uveitis HLA-B27 negative	OU
2	F	43	anterior uveitis HLA-B27 negative	OU
3	F	35	anterior uveitis HLA-B27 negative	OU
4	F	68	anterior uveitis HLA-B27 negative	OU
5	M	39	anterior uveitis assoc. sarcoidosis	OU
6	F	45	anterior uveitis HLA-B27 negative	OU
7	F	30	intermediate uveitis	OU
8	F	26	intermediate uveitis	OU
9	F	33	intermediate uveitis	OS
10	F	36	intermediate uveitis	OU
11	M	32	intermediate uveitis	OU
12	M	32	intermediate uveitis	OU
13	F	57	birdshot chorioretinopathy	OU
14	F	30	birdshot chorioretinopathy	OU
15	F	63	idiopathic panuveitis	OU
16	F	61	panuveitis assoc. sarcoidosis	OU
17	F	65	panuveitis assoc. sarcoidosis	OU
18	M	53	panuveitis assoc. sarcoidosis	OU

*F* Female; *M* Male; *HLA-B27* Human leukocyte antigen B27; *OU* Both eyes; *OS* Left eye

**Table 2:** Cohort description – patients with vitreoretinal lymphoma

Patient	M/F	Age at microbiome sample collection (years)	Age at diagnosis of VRL (years)	Laterality	Vitreous analysis	Age at diagnosis of CNS lymphoma (years)
1	F	82	82	OU	cytology +, FACS +	82
2	F	83	83	OD	cytology +, FACS -	NA
3	M	76	76	OS	cytology +, FACS +	76
4	M	77	77	OU	cytology +, FACS -	NA
5	M	42	42^a^	OU	cytology +, FACS +	36
6	M	67	67	OU	cytology +, FACS -	67
7	M	45	45	OU	cytology +, FACS +	48
8	M	62	62	OU	cytology +, FACS +	62
9	F	67	67	OU	cytology +, FACS +	69
10	M	48	48	OU	cytology -, FACS-	49
11	M	68	68	OU	FACS +	NA
12	F	70	70	OU	cytology +, FACS +	NA
13	F	58	58	OU	cytology -, FACS -	57
14	M	73	73	OU	cytology +, FACS +	NA
15	M	55	55	OU	cytology +, FACS +	NA
16	M	68	68	OU	cytology +, FACS +	NA

*F* Female; *M* Male; *VRL* Vitreoretinal lymphoma; *OU* Both eyes; *OD* Right eye; *OS* Left eye; *FACS* Fluorescence-activated cell sorting; *NA* not applicable

^a^The VRL developed after a five-year remission following CNS lymphoma treatment

### Patients with uveitis showed significant shifts in the oral microbiota

**Fig. 1:**
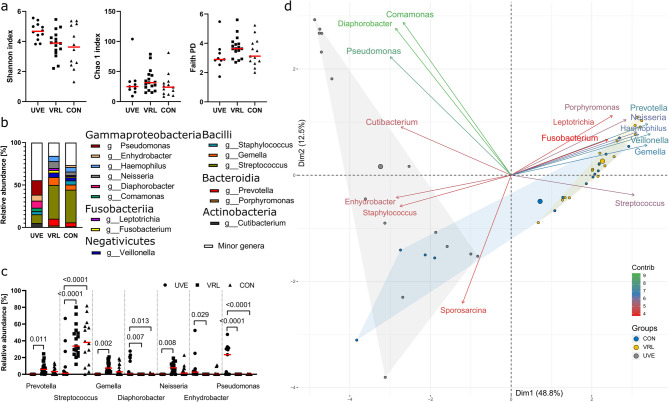
Alpha and beta diversity in buccal swab samples of patients with uveitis (UVE), vitreoretinal lymphoma (VRL) and control subjects (CON). **a** The alpha diversity is described by Shannon diversity index, Chao1 index and Faith phylogenetic diversity (PD). The p-value was calculated by Kruskal-Wallis test with Dunn´s post hoc test for multiple comparisons and the data were presented as medians. **b** Stacked bar chart of microbes at genus level. Microbes with relative abundance higher than 1% are shown in colour. **c** Microbes with significant differences among groups of patients or controls. The p-value was calculated by two-way ANOVA with Tukey´s multiple comparisons test and the data were presented as medians. **d** A principal component analysis biplot of the microbes significantly associated with the group of patients or controls

Oral microbiota composition and function represent factor that can influence regional immune response, including initiation of ocular inflammation or neoplasia. Here, we collected buccal swab samples of patients with uveitis or VRL and healthy control subjects to compare their bacterial communities. We found no significant differences in the buccal microbiota among the groups in terms of alpha diversity, although patients with uveitis or VRL usually showed a tendency towards higher alpha diversity than the control group (Fig. 1A). We observed significant differences in the most frequently detected microbes (Fig. 1B, C). Patients with uveitis showed a significant enrichment of class Gammaproteobacteria, including genera *Pseudomonas*, *Enhydrobacter* and *Diaphorobacter*, compared with lymphoma patients and control subjects. On the other hand, these patients had significantly lower relative abundance of genus *Streptococcus* (class Bacilli) than either control subjects or patients with lymphoma. Patients with VRL showed significantly increased relative abundance of genera *Neisseria* (class Gammaproteobacteria), *Gemella* (class Bacilli) and *Prevotella* (class Bacteroidia) compared to patients with uveitis.

Patient with uveitis had significantly different pattern of the oral microbiota as compared to VRL patients and control subjects (Fig. 1D). In patients with uveitis, the genera *Comamonas*, *Diaphorobacter* and *Pseudomonas* were predominant, whereas in patients with VRL the genera *Prevotella*, *Veilonella*, *Gemella*, *Neisseria* and *Haemophilus* were more common. Additional RDA confirmed that these microbes were significantly associated with the respective group of patients (F(2,43) = 11.907; *p* = 0.0001).

### Difference in the gut microbiota was less expressed

**Fig. 2:**
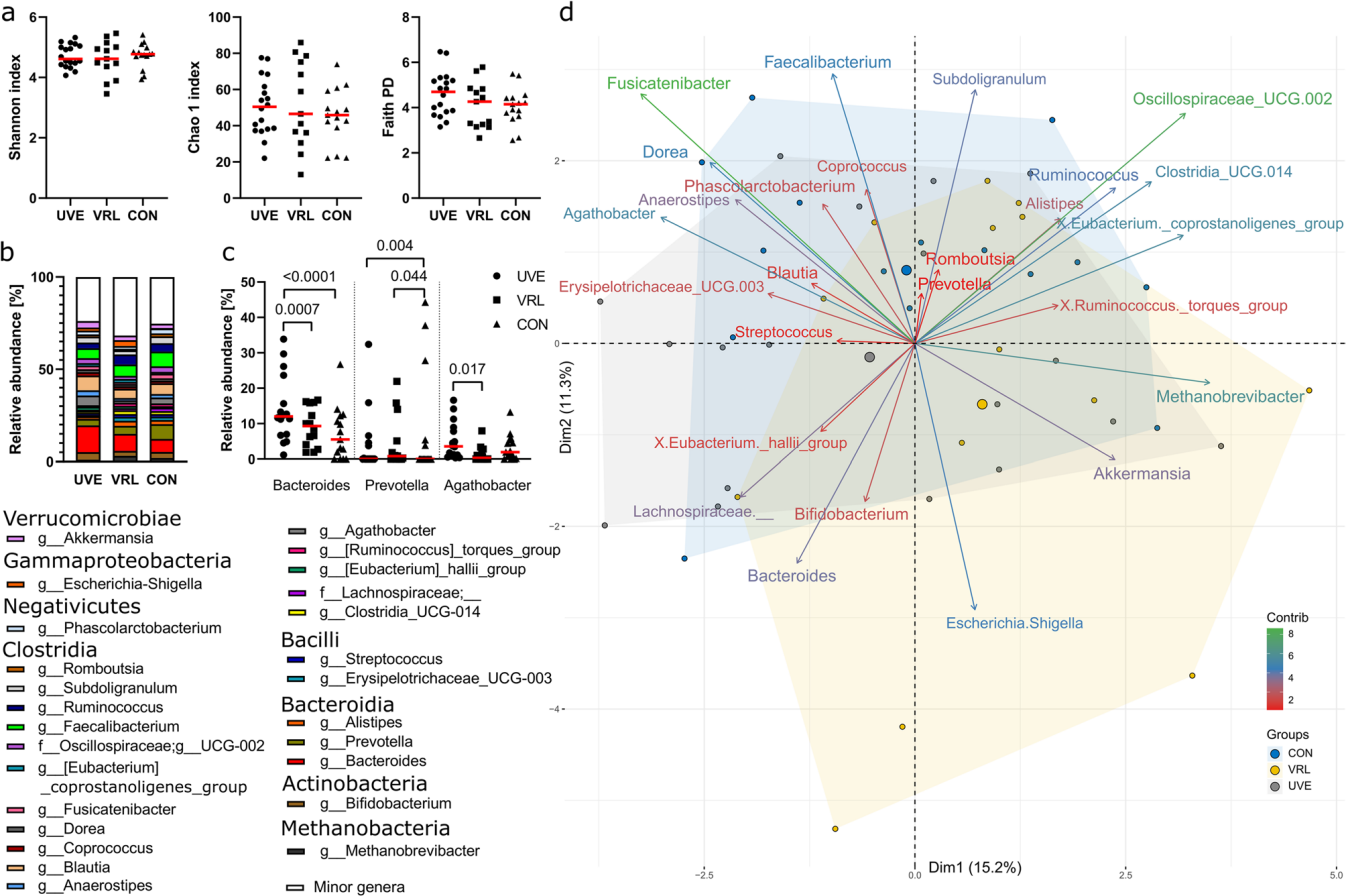
Alpha and beta diversity in faecal samples of patients with uveitis (UVE), vitreoretinal lymphoma (VRL) and control subjects (CON). **a** The alpha diversity is described by Shannon diversity index, Chao1 index and Faith PD. The p-value was calculated by Kruskal-Wallis test with Dunn´s post hoc test for multiple comparisons and the data were presented as medians. **b** Stacked bar chart of microbes at genus level. Microbes with relative abundance higher than 1% are shown in colour. **c** Microbes with significant differences among groups of patients or controls. The p-value was calculated by two-way ANOVA with Tukey´s multiple comparisons test and the data were presented as medians. **d** A PCA biplot of the microbes associated with the group of patients or controls

Next, we compared the faecal microbiota of patients with ocular inflammation, VRL patients and control subjects. We found no significant difference in alpha diversity among groups (Fig. 2A). However, patients with uveitis harboured significantly more members of the genus *Bacteroides* (class Bacteroidia) compared either to patients with VRL or control subjects. Patients with uveitis had also significantly higher abundance of genus *Agathobacter* (class Clostridia) compared with patients with VRL (Fig. 2B, C). Both groups of patients had significantly lower abundance of *Prevotella* (class Bacteroidia) compared to control subjects.

PCA showed greater overlap of the microbes associated with the group of patients or controls (Fig. 2D). Thus, the typical gut microbiota of patients with uveitis was characterized by presence of *Agathobacter* and *Dorea* whereas patients with VRL harboured significantly more of genera *Methanobrevibacter*, *Escherichia*/*Shigella*, *Clostridium_UCG-014* and *Eubacterium coprostanoligenes* group. In control subjects, the genera *Fusicatenibacter*, *Oscillospiraceae_UCG-002*, *Faecalibacterium* and *Subdoligranulum* were enriched in the gut microbiota. These associations were confirmed with the RDA (F(2,44) = 1.467; *p* = 0.034).

## Discussion

The involvement of focal infection in the oral cavity in the aetiology of several inflammatory diseases, including uveitis, was the reason not only for dental examinations but also for radical treatment procedures in the first half of the 20th century [42]. This historical perspective suggests a potential causal relationship where local microbiota may be involved. Moreover, in the endemic areas (i.e. Turkey, Japan, Korea), a reduction in the incidence and severity of BD has been observed with an improvement in oral health [22, 38–41]. Since specific shifts in gut microbiota are associated with spondylarthritis and associated diseases [21, 53, 54], we excluded the patients with BD-associated uveitis or HLA-B27 positive anterior uveitis from our study.

The oral microbiota is the second most diverse microbial community with higher alpha-diversity but lower beta-diversity when compared to other body sites. And although the oral microbiota is more prone to changes as it easily respond to factors such as food, drinks and oral hygiene, we believe that it better reflects the diseases of head and neck, especially, as these areas share the cervical draining lymph nodes, where the immune reaction is initiated and further sustained. In our study, we found no changes in alpha diversity among groups, but there were uveitis- and VRL-specific shifts in the beta diversity. We found significant enrichment of genera *Pseudomonas*, *Enhydrobacter* and *Diaphorobacter* that was accompanied by significant reduction of *Streptococcus*, *Prevotella*, *Gemella* and *Neisseria* in the oral microbiota of patients with uveitis. The oral microbiota is rarely studied in patients with uveitis, but there are several studies on the oral microbiota in BD. High abundance of *Streptococcus mutans* and dysbiosis are associated with the oral microbiota of BD patients [55] and higher abundance of *Streptococcus* and lower abundance of *Veillonella* is even associated with ulcers in BD [56]. Joubert et al. pointed out changes in the oral microbiota, where *Streptococcus sanguinis* is preferentially associated with oral aphthous ulcerations, consistent with a suspicion of pathogenesis through molecular mimicry [22]. Together, our results suggested that potential pathogens or microbes not commonly found in the oral cavity were promoted while oral commensals were reduced at the same time [57, 58].

The oral microbiota of patients with VRL showed significantly increased relative abundance of the genera *Neisseria*, *Gemella* and *Prevotella* compared to patients with uveitis but not compared to controls. All these microbes are the most abundant members of healthy oral microbiota [59]. In addition, VRL patients and controls had a significantly increased relative abundance of the genus *Streptococcus*. Although it appears that only changes in the proportions of resident commensals characterize the oral microbiota of patients with VRL, some of these microbes, such as *Gemella*, *Prevotella* and *Streptococcus*, may possess virulence factors that can promote neoplastic transformation of cells [60, 61].

The analysis of gut microbiota composition in our cohorts found no difference in the alpha diversity compared to healthy controls, only a minor reduction in Shannon index of patients with VRL which is in line with findings of the analysis of gut microbiota in HLA-B27 positive and HLA-B27 negative patients with anterior uveitis [27] and in systemic lymphomas [32, 33]. Although some studies reported significantly lower alpha diversity in stool samples from treatment-naïve, newly diagnosed patients with systemic lymphoma compared to healthy controls [31, 36].

Next, we found that patients with uveitis harboured significantly more members of genus *Bacteroides* and lower abundance of *Prevotella* compared to control subjects. Gut microbiota of our cohort with uveitis was characterized by presence of genera *Agathobacter* and *Dorea*. While other study has shown reduction of *Dorea* in the gut microbiota of patients with BD as well as in patients with VKH. Taken together, they observed lower abundance of SCFA-producing microbes from the class Clostridia and genus *Erysipelotrichaceae* in patients with VKH and BD [54]. Analysing faecal samples from patients with BD, lower levels of butyrate-producing bacteria of *Clostridium* spp. and methanogens of *Methanoculleus* spp., *Methanomethylophilus* spp. have been reported [25]. Kalyana Chakravartha et al., who analysed gut microbiota from 13 patients with various types of uveitis, has shown reduced diversity of several “anti-inflammatory” microbes, including *Faecalibacterium*, *Bacteroides*, *Lachnospira*, *Ruminococcus*, and enrichment of “pro-inflammatory” ones, such as *Prevotella* and *Streptococcus* [28]. Similarly, the existence of a common gut dysbiosis in spondylarthritis and related inflammatory pathologies, including acute anterior uveitis has been observed [53]. In a recent case control study, Morandi et al. analysed gut microbiota composition in patients with HLA-B27-associated non-infectious anterior uveitis, HLA-B27-negative controls, and HLA-B27-positive healthy controls. They found significantly higher levels of the *Eubacterium ramulus* species in HLA-B27-negative controls. An additional analysis showed an increase of the species *Phocaeicola vulgatus* in active HLA-B27-positive anterior uveitis compared to HLA-B27-negative controls, as well as a decrease of the species *Bacteroides caccae* compared to HLA-B27-positive controls [21]. This is in line with our finding that control subjects had higher abundance of several SCFA producers.

Since no results of gut microbiota analysis in patients with VRL have been published so far, we compared our findings with the results of microbiota analyses in systemic LBCL. In the patients with VRL, we observed higher abundance of genus *Bacteroides* while *Prevotella* was reduced. In line with our study, an increase in Bacteroidota with a concomitant decrease in Firmicutes at the phylum level was shown in patients with lymphoma [31] while other studies demonstrated that the abundance of genus *Bacteroides* was significantly lower in patients with LBCL [32, 33]. In addition, we found increased abundance of *Escherichia*/*Shigella* although it was not statistically significant when compared to the control subjects. Nevertheless, our cohort of patients with VRL specifically harboured several genera, including *Escherichia*/*Shigella*, *Methanobrevibacter*, *Clostridium_UCG-014* and *Eubacterium coprostanoligenes* group. Yoon et al. identified greater abundance of facultatively anaerobic species, especially from Enterobacteriaceae family, including *Escherichia coli*, *Citrobacter freundii* and *Enterobacter faecium* [36]. Moreover, significantly higher abundance of genus *Escherichia*/*Shigella* was reported in the patients with LBCL compared to the control group [32, 33]. Taken together, patients with lymphoma seem to have specific pattern of the gut microbiota with reduced capacity to produce SCFA and increased presence of pathobionts, such as *Escherichia*/*Shigella*.

We are aware of several factors that can limit the outcomes of our study. The age of the control group has very wide range given by uveitis patients being younger than patients with lymphoma. We also grouped all anatomical types of uveitides into one group although they might be related to different immunopathological mechanisms. We used 16S rRNA sequencing based-approach that do not include the information on microbial metabolism, which may differently affect the disease. We did not collected data on the status of oral health or additional medication, nevertheless, we supposed that these factors are age-related and distributed in patients and controls equally. Despite these limitations, we believe that our study brings new and important knowledge of oral and gut microbiota in patients with uveitis and VRL.

## Conclusion

To the best of our knowledge, our study, as the first one, highlighted significant differences in the composition of oral microbiota among patients with uveitis, vitreoretinal lymphoma, and control subjects. While, in the oral microbiota, the commensal strains with pathogenic potential were enriched in patients with uveitis and VRL, in the gut microbiota, no clear pattern was observed. Nevertheless, further studies are needed to clarify whether the microbial shifts are linked to specific pathways leading to manifestation of these diseases. Finally, deeper knowledge of the pathogenetic mechanisms may lead to development of new therapeutic approaches.

## Data Availability

The datasets used and analysed during the current study are available from the corresponding author on reasonable request. Raw data from 16S rRNA sequencing are available at the National Center for Biotechnology Information (NCBI) in the Sequence Read Archive (SRA) at http://www.ncbi.nlm.nih.gov/sra under the accession number PRJNA1081169.
